# Cowpox with Severe Generalized Eruption, Finland

**DOI:** 10.3201/eid0911.020814

**Published:** 2003-11

**Authors:** Paula M. Pelkonen, Kyllikki Tarvainen, Arja Hynninen, Eva R.K. Kallio, Heikki Henttonen, Airi Palva, Antti Vaheri, Olli Vapalahti

**Affiliations:** * Faculty of Veterinary Medicine, Helsinki, Finland; † Haartman Institute, Helsinki, Finland; ‡ Central Hospital of North Karelia, Joensuu, Finland; § Finnish Forest Research Institute, Vantaa, Finland; ¶ HUCH Laboratory Diagnostics, Helsinki, Finland

**Keywords:** Cowpox, diagnosis, epidemiology, orthopoxvirus, phylogeny, seroprevalence, zoonoses

## Abstract

Cowpox with a severe, generalized eruption was diagnosed in an atopic 4-year-old girl by electron microscopy, virus isolation, polymerase chain reaction, and immunoglobulin (Ig) M and low-avidity IgG antibodies. The hemagglutinin gene of the isolate clustered with a Russian cowpox virus strain, and more distantly, with other cowpox and vaccinia virus strains. The patient’s dog had orthopoxvirus-specific antibodies, indicating a possible transmission route.

Cowpox is a zoonotic dermatitis affecting, despite its name, mainly cats and humans. The disease is caused by cowpox virus, a close relative to vaccinia, smallpox (variola), and monkeypox viruses within the *Orthopoxvirus* genus (*1*). The relationship between cowpox and vaccinia viruses has been unclear since Edward Jenner used a virus isolate from cows for smallpox vaccination (*2*,*3*). Orthopoxviruses, comprising a genus in the family *Poxviridae,* are large, brick-shaped viruses with a 200-kbp DNA genome, and they replicate in the cytoplasm (*4*). Because immunity to orthopoxviruses is cross-reactive, smallpox vaccination might have suppressed cowpox virus infections in the human population. Cowpox virus is not highly infective for humans and usually produces a localized lesion mainly on fingers, hands, or face (*5*). In immunocompromised persons, however, the disease may lead to death (*6*). The virus infects through skin abrasions, resulting in successive lesions of macular, papular, vesicular, pustular, ulceral, and eschar stages for 2 weeks. Systemic symptoms are also common (*5*). The reservoir hosts of cowpox are wild rodents (*7*); wild rodents may transmit the virus to humans through cats (*5*) or other pets that roam outside. Direct transmission from a rodent to a girl has been recently described (*8*). Both cowpox and monkeypox, which was recently transmitted to the United States by transport of animals indigenous to Africa (*9*), are actually orthopoxviruses of their reservoir rodents and are not well adapted to interhuman spread (*1*). The misleading nomenclature is based on the hosts from which they were first identified (*1*). Cowpox virus infections have been detected in Europe and central and northern Asia (*1*). Nongeneralized infections in children have been previously characterized and diagnosed by electron microscopy (*10*), virus isolation, polymerase chain reaction (PCR), and restriction enzyme analysis (*10*,*11*).

## The Study

**Figure 1:**
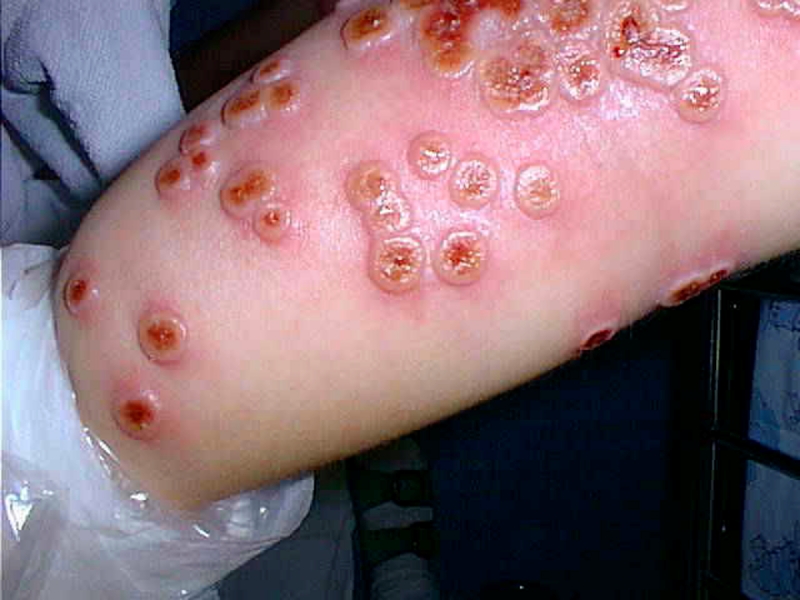
Cowpox lesions on patient’s forearm on day 7 after onset of illness.

A 4-year-old girl from a small farm in eastern Finland was hospitalized in September 2000 because of umbilicated vesicopapules, which developed over the previous 5 days (Figure 1), and unresponsiveness to cephalexin. She had a past history of moderate atopic dermatitis. Animals at her home farm included a horse, three dogs, and a rabbit, but she had no contact with cats because of allergy. On examination, most lesions were located on her swollen red extremities, a few were found on the side of her body, and 3-mm lesions were found on the face and vulva. All lesions were at the same stage of development. She was febrile with 38°C temperature and feeling unwell. A biopsy sample from a papule was sent to a virology laboratory, where orthopoxvirus particles 230 × 300 nm in size were demonstrated by electron microscopy with negative staining.

The girl was treated in isolation at the hospital with chlorhexidine washings and wound dressings with fusidic acid. Intravenous dicloxacillin was administered to prevent secondary bacterial infections. On day 12, skin lesions progressed to deep-seated, hard, black eschars. At the same time the patient’s general condition improved. Two months later, all lesions were healed with scars.

**Figure 2:**
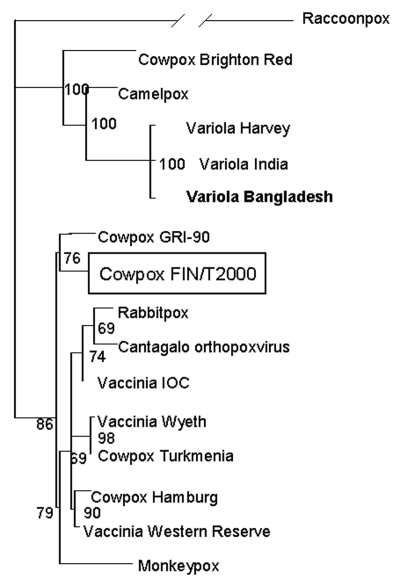
Phylogenetic tree of selected orthopoxvirus hemagglutinin genes based on Clustal X alignment and the maximum likelihood method TreePuzzle. Virus sequences used for the analysis were raccoonpox as an outgroup (GenBank accession no. M94169); cowpoxvirus strains Brighton Red (AF482758), FIN/T2000 (AY366477), GRI-90 (Z99047), Hamburg (Z99050), and Turkmenia (Z99048); vaccinia virus strains IOC (AF229248), Western Reserve (M93956), and Wyeth (Z99051); variola virus strains Bangladesh (L22579), Harvey (X65516) and India (X69198); camelpox virus (AF438165); Cantagalo orthopoxvirus (AF229247); monkeypox virus (Z99049); and rabbitpox virus (Z99049).

A homogenized biopsy sample was used to infect Vero cells, where a cytopathic effect typical of orthopoxviruses was seen after 2 days. The isolate, designated CPXV/FIN/T2000, was shown to contain orthopoxvirus by electron microscopy, and DNA samples from both the infected cells and the original biopsy specimen were PCR-positive for orthopoxvirus thymidine kinase gene (*12*). The hemagglutinin (HA) gene of the isolate was amplified (*13*), sequenced (948 nucleotides; accession no. AY366477), and compared to other orthopoxviruses. The CPXV/FIN/T2000 strain differed 3% to 4% at the nucleotide level from cowpox virus strains available in GenBank. The sequences were further subjected to phylogenetic analysis: they were aligned by using Clustal X with Gonnet protein matrix and analyzed by using the maximum likelihood phylogenetic software TREE-PUZZLE 5.0, applying the Hasegawa model of substitution and performing 25,000 steps. The HA sequence of CPXV/FIN/T2000 formed a separate clade with cowpox virus strain GRI-90 (Figure 2), isolated originally from another 4-year-old girl, who contracted cowpox after playing with a mole near Moscow (*14*). The reference cowpox virus strain Brighton grouped with camelpox and variola viruses instead of other cowpox or vaccinia viruses. However, because of the high homology of HA genes, this finding should be interpreted with caution. The remaining cowpox virus strains clustered together with vaccinia viruses; this clustering may, in some cases, be explained by origin from vaccinia virus strains that had escaped to nature (*13*). This finding seems not to be the case in Finland, since the HA nucleotide sequence of vaccinia virus used in Finland differed 4% both from the strain CPXV/FIN/T2000 and another Finnish cowpox virus isolate from 1989 with an identical HA sequence (data not shown). Different alignment parameters and the neighbor-joining method produced the same results with high support values. Scattered phylogenetic distribution of cowpox virus strains is supported by data presented by other researchers and may reflect an ancestral role of cowpox viruses within the *Orthopoxvirus* genus; some strains cluster with vaccinia viruses and others (including the reference Brighton strain) with variola virus (*1*).

An immunofluorescence assay (IFA) to measure specific immunoglobulin (Ig) G, IgM, and avidity of IgG antibodies (*15*) was established on acetone-fixed, CPXV/FIN/T2000-strain-infected Vero cells. The patient’s serum had a high IgM-antibody titer at admission and low avidity of specific IgG; after 60 days, the IgM level was low and the IgG avidity high (Table 1).

**Table 1:** Orthopoxvirus antibodies in sera of the patient and her pets^a^

Characteristic	Titer		
	IgM	IgG	IgG avidity (%)
Patient day 7	1280	2560	1.5
Patient day 14	640	2560	3.1
Patient day 60	10	640	25
Old-immunity controls (>50 y)	<10	80–640	50–100
Negative controls	<10	<10	—
Dog A	—	<20	—
Dog B	—	320	50
Dog C	—	20	50
Horse	—	<20	—
Rabbit	—	<20	—

^a^IG, immunoglobulin.

The serum samples from the patient’s pets were collected later and tested with IFA. A hunting dog had antibodies with a titer of 320; another dog had a titer of 20. Thus, a dog might have transmitted the infection from a wild rodent to the patient, although cats are thought to be the main source of human infection (*5*).

We further studied orthopoxvirus antibodies by IFA in Finnish fauna and humans. The seroprevalence rates for cats and horses were 3.9% and 1.6%, respectively; the rates for wild rodents (when either serum or lung or heart extracts was used) were 0% to 92%, depending on the trapping time and the location (Table 2). In Värtsilä, a rural district in eastern Finland near where the patient lives, antibodies were found in 1 (2.8%) of 36 rodents. However, variation of seroprevalence rates in rodents is also influenced by population dynamics: low prevalences are often found in the increase phase and high prevalences in the peak phase of rodent population fluctuations. The positive rodents were mainly bank voles *(Clethrionomys glareolus*)*.* The two seropositive horses were from a region near where the patient lives in eastern Finland, and the three seropositive cats were free-roaming and found in southern Finland. In addition, seroprevalence rates of 50% (7/14) and 1.4% (1/73) have been found in foxes and lynxes, respectively, from a limited geographic area in Finland (*16*).

**Table 2:** Orthopoxvirus antibodies in cat, horse, and wild rodent panels, Finland

Panel	n	Positive	Prevalence (%)
Cats	77	3	3.9
Horses	127	2	1.6
Wild rodents			
Southern Finland: Evo	36	33	91.7
Eastern Finland: Värtsilä	36	1	2.8
Western and central Finland:			
Several localities	436	0	0
Lapland: several localities	394	7	1.8 (0.4–15.2)
Wild rodents altogether	902	41	4.5

Sera collected at a Finnish Veterinary meeting in 2001 showed that every person >50 years had orthopoxvirus antibodies, as measured by IFA. The seroprevalence decreased gradually for younger age groups (Table 3), reflecting the gradual cessation of smallpox vaccination (the last vaccinations in Finland occurred in 1977). The average population might have lower seroprevalence rates than veterinarians because of veterinarians’ frequent contact with animals that may harbor orthopoxvirus. Consequently, younger age groups are more susceptible to smallpox and cowpox virus infections.

**Table 3:** Orthopoxvirus antibodies in humans (veterinarians), Finland

Humans	n	Positive	Prevalence (%)
<25 y	19	2	10.5
26–30 y	23	4	17.4
31–50 y	78	46	59.0
>51 y	18	18	100.0

Although cowpox virus infection usually causes a single, painful, ulcerated vesicopustule and local lymphadenopathy, immunocompromised patients and children, especially those with atopic eczema, are susceptible to a generalized, even lethal, smallpoxlike infection (*5*,*6*). An early diagnosis and prompt recognition of the virus are essential for treating and differentiating cowpox, from other orthopoxvirus and herpesvirus infections, especially in severe cases.

## Conclusions

Cowpox virus (orthopoxvirus) infection was diagnosed by electron microscopy, PCR, virus isolation, and serologic testing (positive IgM or low avidity of IgG antibodies). Cowpox virus strains show considerable genetic variations with different positioning in the orthopoxvirus phylogenetic tree. Circulation of cowpox virus in wild and domestic animals, together with decreased immunity in humans, may lead to increased occurrence of human cowpox, especially in atopic and immunocompromised persons who are at risk for generalized infection. The described case further suggests atopy to be a contraindication to smallpox vaccination.
